# Nerve Growth Factor from Cobra Venom Inhibits the Growth of Ehrlich Tumor in Mice

**DOI:** 10.3390/toxins6030784

**Published:** 2014-02-26

**Authors:** Alexey V. Osipov, Tatiana I. Terpinskaya, Elena V. Kryukova, Vladimir S. Ulaschik, Lubov V. Paulovets, Elena A. Petrova, Ekaterina V. Blagun, Vladislav G. Starkov, Yuri N. Utkin

**Affiliations:** 1 Shemyakin-Ovchinnikov Institute of Bioorganic Chemistry, Russian Academy of Sciences, ul. Miklukho-Maklaya 16/10, Moscow 117997, Russia; E-Mails: evkr@mail.ru (E.V.K.); vladislavstarkov@mail.ru (V.G.S.); utkin@mx.ibch.ru (Y.N.U.); 2 Institute of Physiology, National Academy of Sciences of Belarus, ul. Akademicheskaya, 28, Minsk 220072, Belarus; E-Mails: terpinskayat@mail.ru (T.I.T.); biblio@fizio.bas-net.by (V.S.U.); primabsu@yandex.ru (L.V.P.); Helena_iseu@mail.ru (E.A.P.); Kate_blagun@mail.ru (E.V.B.)

**Keywords:** Ehrlich carcinoma, K252a, nerve growth factor, TrkA receptor, MCF-7 cell line

## Abstract

The effects of nerve growth factor (NGF) from cobra venom (cvNGF) on growth of Ehrlich ascites carcinoma (EAC) cells inoculated subcutaneously in mice have been studied. The carcinoma growth slows down, but does not stop, during a course of cvNGF injections and restores after the course has been discontinued. The maximal anti-tumor effect has been observed at a dose of 8 nmoles cvNGF/kg body weight. cvNGF does not impact on lifespan of mice with grafted EAC cells. K252a, a tyrosine kinase inhibitor, attenuates the anti-tumor effect of cvNGF indicating the involvement of TrkA receptors in the process. cvNGF has induced also increase in body weight of the experimental animals. In overall, cvNGF shows the anti-tumor and weight-increasing effects which are opposite to those described for mammalian NGF (mNGF). However in experiments on breast cancer cell line MCF-7 cvNGF showed the same proliferative effects as mNGF and had no cytotoxic action on tumor cells *in vitro*. These data suggest that cvNGF slows down EAC growth via an indirect mechanism in which TrkA receptors are involved.

## 1. Introduction

Nerve growth factor (NGF) is a neurotrophin participating in processes of growth, differentiation, and survival of neurons both in the peripheral and the central nervous systems [1]. Secreted protein regulators neurotrophins which control neuronal development, survival, and activity, are members of a larger family of growth factors. Being ubiquitous in animal kingdom, neurotrophins are found both in vertebrates and invertebrates. Although being very important for the maintenance of central and peripheral nervous systems, they possess several critical roles outside the nervous system, for example, in kidney and cardiac development, spermatogenesis, and regulation of immune system and skin [2,3]. In mammalians, very different tissues and organs can secrete NGF (for examples, salivary glands, skin, cardiomyocytes, testis and epidydimus, as well as cells and organs of the immune system [4]) and different organs express receptors for NGF and other neurotrophins [2]. Interestingly, NGF is present in noticeable (up to 0.5%) amounts in snake venom [5]. NGF synthesized by venomous glands of snakes is similar to mammalian NGF (mNGF) structurally and functionally, but it can be easier isolated from natural source (snake venom) in a native form. Although recombinant mNGF is on the market, it is extremely expensive.

Neurotrophins exert their action through the specific receptors - tropomyosin-related kinase (Trk) subfamily of receptor tyrosine kinases. In mammals, these receptors are presented by the three members and exhibit ligand selectivity: NGF is the preferential high-affinity ligand for TrkA. However, it is also a ligand for p75 neurotrophin receptor (p75NTR), a member of the tumor necrosis factor receptor superfamily, that binds all neurotrophins with similar affinities, regulates apoptosis and has no tyrosine kinase activity (see review [6]). Expression of TrkA and p75NTR has been shown not only in neuronal cells, but also in cells of different origin, including breast cancer cells [7].

Trk tyrosine kinase activity leads to stimulation of the mitogen-activated protein kinase cascade. The Trk subfamily of receptors was shown as an important player in carcinogenic progression in non-neuronal tissues. Breast tumors present increased levels of TrkA and phospho-TrkA. Overexpression of TrkA enhances tumor growth, angiogenesis and metastasis of xenografted breast cancer cells in immunodeficient mice [8]. mNGF, being a TrkA ligand, is able to stimulate the proliferation of breast cancer cell lines *in vitro* and *in vivo* in immunodeficient mice [9]. On the other hand, in human, enhanced levels of TrkA expression correlate with survival of patient with breast cancer [10].

mNGF has been shown to be involved in promoting the tumor growth via perineural invasion (in pancreatic and oral cancers) [11,12], via an autocrine loop (in breast and prostate cancer) [13], via activating angiogenesis [14] as well as in progression of some other cancer symptoms [15]. On the other hand, mNGF suppresses human prostate tumor growth in nude mice [16] as well as human leukemia K562 cell proliferation *in vitro*, and administration of TrkA agonist, NGF mimetic gambogic amide, inhibited K562 cell proliferation in nude mice [17].

Thus, further *in vivo* investigations are required to clarify role(s) of NGF and its receptors in development and growth of non-neural tumors. Recently we have reported in a short communication that cobra venom NGF (cvNGF) can suppress Ehrlich ascites carcinoma (EAC) cell growth [18]. In this paper we present the detailed studies of cvNGF effects on growth of EAC cells *in vivo* and show the involvement of TrkA receptors in the suppression process.

## 2. Results and Discussion

**Figure 1:**
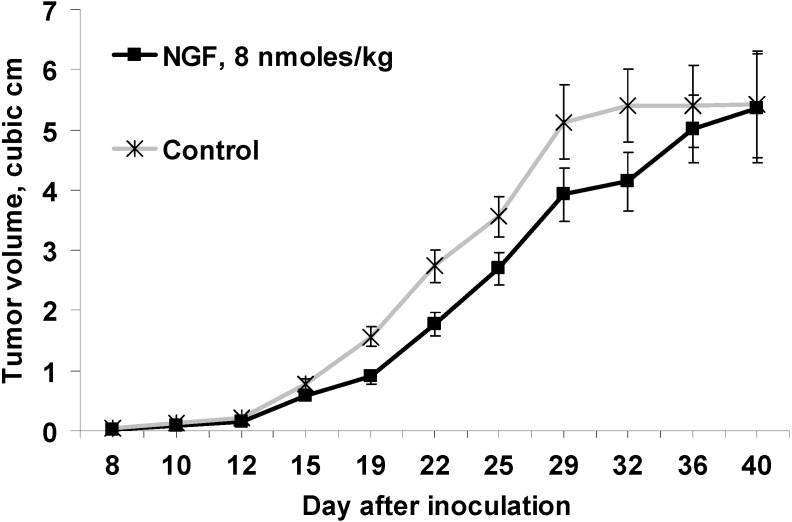
Effect of cobra venom nerve growth factor (cvNGF) on growth of Ehrlich ascites carcinoma (EAC) inoculated subcutaneously in mice, a 32-day treatment with cvNGF. Mice were inoculated with 6 million EAC cells subcutaneously, in 15–20 min they received cvNGF intraperitoneally at dose of 8 nmol/kg body weight. The injections were done intraperitoneally every 3–4 days 10 times during 32 days, *n =* 15. The response of EAC to 32-day treatment with cvNGF; *p* (NGF-Control) < 0.001 as ANOVA.

Ehrlich carcinoma is a cultivated tumor cell line originated from spontaneous breast cancer. The existing data about NGF influence on the breast cancer are controversial: on the one hand, it has been shown that mNGF stimulates the proliferation of breast cancer cell lines [9], while on the other the elevated level of NGF receptor Trk-A correlates with survival of patient with breast cancer [10]. To address this problem, the mice with grafted EAC were treated with cvNGF. The cvNGF for this study was isolated from cobra venom using three different types of liquid chromatography [19]. Its purity checked by analytical reversed phase HPLC and MALDI mass-spectrometry was 98%. Several series of experiments were carried out. When cvNGF was injected alone, a reliable decrease in subcutaneous EAC growth *in vivo* was observed during the course of cvNGF injections. In the experiment 1, the volume of subcutaneous solid tumor was about 60%–70% of control (*i*.*e*., 30%–40% inhibition) from day 15 to day 32 after inoculation (Figure 1). The decrease was statistically significant during all this period as shown by the ANOVA analysis (*p <* 0.001). However, the effect disappeared rapidly within several days after discontinuation of cvNGF injections at day 32. Despite the retardation of tumor growth during the injection course and shortly after it, lifespan of experimental mice increased only to a small extent: lifetime median (1st ÷ 3d quartile) is 49.0 (36.5 ÷ 55.5) days for cvNGF treated mice *vs*. 40 (34.5 ÷ 50.5) days for control, *p >* 0.05. The trends in the reversibility of the effect and insignificance of lifespan change were repeatedly observed in the next experiments too.

**Figure 2:**
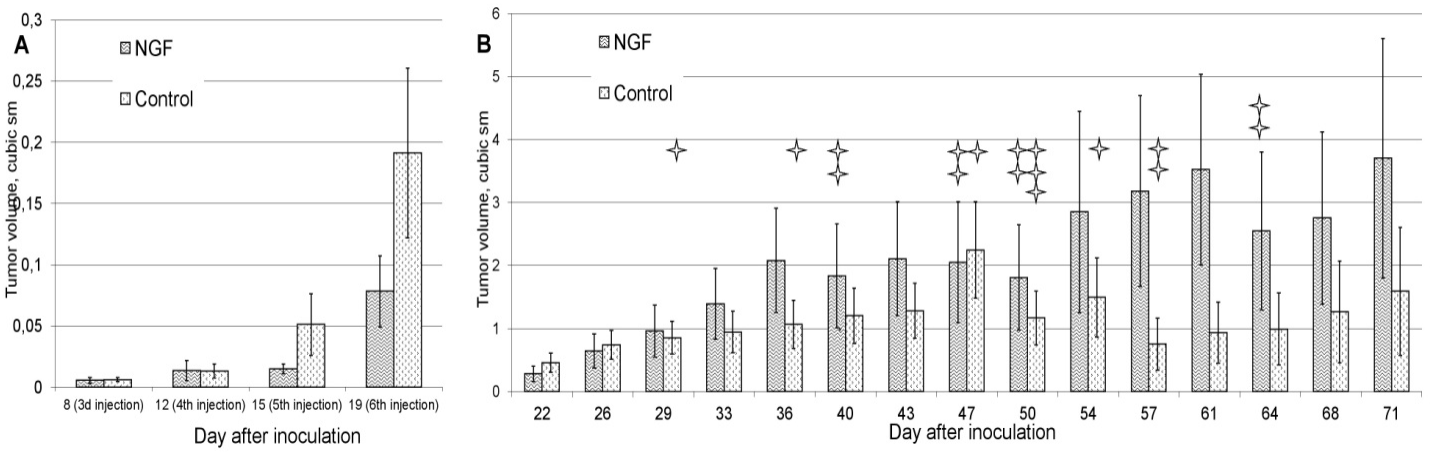
The response of EAC to 19-day treatment with cvNGF (as tumor size per living animal): (**A**) during course of NGF treatment; (**B**) after the course of cvNGF treatment. Mice were inoculated with 6 million EAC cells subcutaneously, in 15–20 min they received cvNGF intraperitoneally at dose of 8 nmol/kg body weight. Then, the injections were done intraperitoneally every 3–4 days 6 times during 19 days, *n =* 10. *p* (NGF-Control) > 0.05 as ANOVA for A and B. No significant difference is observed, there is a trend only. Cases of deaths are signed by asterisks.

In the experiment 2, on the day 19, when the trend to tumor growth suppression by cvNGF had already developed showing 60% inhibition (Figure 2A), cvNGF injections were discontinued, and mice were observed for several weeks more. Surprisingly, tumors in treated mice overtook those in the control group in one week and then surpassed them by 1.5–2-fold and even more at certain stages (Figure 2B). It should be noted that if a mouse in any group dies, the mean tumor volume in living animals within the group may change. The mice deaths are shown by asterisks in Figure 2B. Given that, as a rule, such a mouse developed great tumor, the mean tumor volume per living animal was reduced in the particular series.

In contrast to the above data for the subcutaneous inoculation, cvNGF had no effect on growth of EAC inoculated intraperitoneally in mice (3 injections for 8 days; further injections were hindered by rapid development of intraperitoneal ascitic tumor; data not shown). It was unexpected to some extent, as cvNGF was injected intraperitoneally too, that is in close vicinity to tumor. The reason for this discrepancy might be in the fact, that Ehrlich carcinoma grew rapidly in peritoneum and mice lifetime was only 19–25 days after transplantation. However, the cvNGF antitumor effect might take a longer time to develop: more than 15 days, as it was at subcutaneous inoculation.

As mentioned in Introduction, NGF binds the two types of receptors (TrkA and p75NTR) mediating its biological activities with different affinities. To understand what type of the receptor is involved in the observed NGF effects, different cvNGF doses were tested and effects of K252a were examined. K252a is an inhibitor of tyrosine kinases displaying selectivity to Trk subfamily.

**Figure 3:**
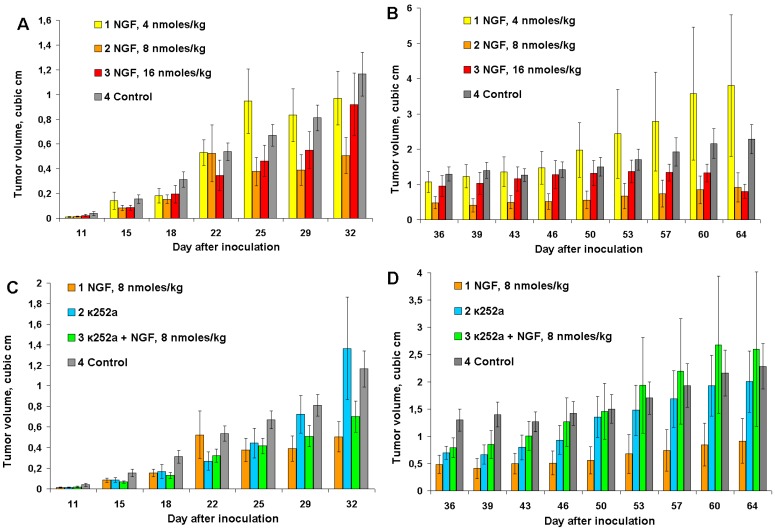
The response of Ehrlich carcinoma to cvNGF injected at concentrations 4, 8, and 16 nmoles/kg (**A**,**B**) and to combined treatment with cvNGF and k252a (**C**,**D**): (**A**,**C**) the injections were done 10 times during 32 days (every 3–4 days); (**B**,**D**) after termination of injections at day 32. *p* (column 2, 3, *vs*. column 4) < 0.001; *p* (column 1, *vs*. column 4) > 0.05; as ANOVA for (**A**,**B**). *p* (column 1, 2 *vs*. column 4) < 0.001; *p* (column 2 *vs*. column 4) > 0.05; as ANOVA for (**C**). *p* (column 1, 2 *vs*. column 4) < 0.001; *p* (column 3 *vs*. column 4) > 0.05 as ANOVA for (**D**).

In cvNGF concentration dependence experiments, mice were inoculated with 1 million of EAC cells instead of 6 million used in the previous experiments. This results in a slower tumor development. Three different cvNGF doses were tested: 4, 8 and 16 nmoles/kg. The anti-tumor effect of cvNGF was maximal at the dose of 8 nmoles/kg; it was less noticeable at 16 nmoles/kg and statistically insignificant at 4 nmoles/kg (Figure 3A, columns 2, 3, and 1, respectively). After termination of cvNGF treatment, tumor volumes in mice which received 8 nmoles of cvNGF per kg of body weight remained smaller as compared to other groups (Figure 3B). Interestingly, in contrast to our previous experiments (Figure 1 and Figure 2), a long-lasting anti-tumor cvNGF effect at dose of 8 nmoles/kg was observed if mice were of inoculated with one million of carcinoma cells. However, again no improvement in mice survival was found up to day 90 of the experiment (data not shown).

As no data about the K252a effects on Ehrlich carcinoma can be found in the literature we have studied its influence on EAC growth. To understand the effects of K252a, it was administered to mice both with and without cvNGF. It was found that K252a injected alone retarded *in vivo* Ehrlich carcinoma growth to some extent (e.g., days 15 to 25 and 36 to 46, Figure 3C,D, column 2 *vs*. control 4), which is in agreement with data of [8]. It should be noted that the anti-tumor effect of K252a was smaller than that of cvNGF (Figure 3C,D, column 2 *vs*. column 1). Such an *in vivo* K252a activity may be related either to the inhibition of oncogenic form(s) of some tyrosine kinase receptors [20,21] or to its angiostatic properties [22]. Against expectations, cvNGF and K252a did not amplify the anti-tumor effect of each other. Moreover, simultaneous administration of cvNGF and K252a resulted in suppressing of anti-tumor effects of both cvNGF (by about 2-times—Figure 3C,D, column 3 *vs*. column 1) and, to a lesser extent, K252a (Figure 3C,D, column 3 *vs*. column 2). These results may be explained by competitive action of cvNGF and K252a on the same TrkA receptor(s) and therefore anti-tumor cvNGF activity is related more probably to its action on TrkA rather than p75NTR receptors. 

It was shown [13] that two different Trk receptors can induce both differentiation and proliferation within the same cell, and several tyrosine kinase receptors were found to be overexpressed in breast cancer cells [23]. It was also observed that TrkA cooperates with HER2 to activate breast cancer cell proliferation under mNGF stimulation [24]. Therefore, one may suggest that two or more different Trk-receptors (or even other tyrosine kinase receptors), not only TrkA, responding to K252a are involved in the process of Ehrlich carcinoma growth.

It is well-known that activation of p75NTR by neurotrophins induces apoptosis in tumor cells of neuronal origin. mNGF is also able to trigger *in vitro* apoptosis via p75NTR in cells of other origins, for examples, myofibroblasts [25] or melanoma cells [26]. However, concerning breast cancer, p75NTR overexpression brings resistance to apoptosis and favors tumor growth both *in vitro* and *in vivo* as shown in an immunodeficient mice xenograft model [27]. In our particular model it is hardly possible that cvNGF suppresses EAC growth through p75NTR.

At present a great deal of data exists about dependence of tumor ability to enhance or to reduce proliferation on a level of expression and functionality of NGF-binding receptors [6,13]. For example, in neuroblastoma, increased level of TrkA expression has a favorable prognosis for spontaneous regression or differentiation [28]. TrkA activation inhibits proliferation of human leukemia cells *in vitro* and *in vivo* in mice [17]. At the same time, most of data shows that TrkA activation, including that by mNGF, stimulates progress of breast tumor ([8,9] and many other). Only few reports show the opposite NGF effect. For example, enhanced levels of TrkA expression in samples of human breast cancer correlated with increased rate of patient survival [10]; authors hypothesized that tumors with high levels of TrkA receptors had retained some physiological control of growth, which could explain the better prognosis.

It should be noted that there are no data both on the TrkA receptors in Ehrlich carcinoma and the effects of NGF on this tumor. It was shown only that in mice the incubation of Ehrlich tumor cells with mouse submaxillary salivary gland extract before inoculation resulted in the tumor reduction of about 30% in size [29]. As submaxillary salivary glands are rich source of mNGF, this result support our finding.

**Figure 4:**
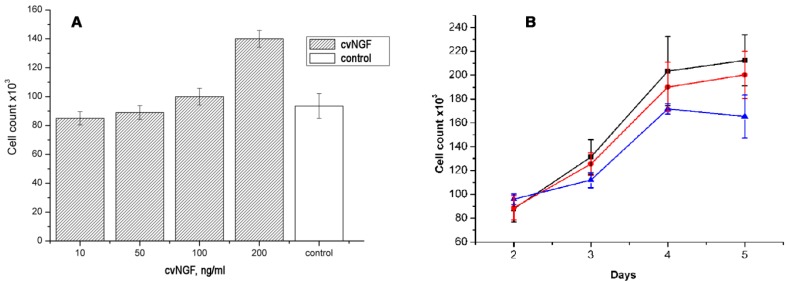
NGF effects on the proliferation of MCF-7 breast cancer cell line. (**A**) The cells were incubated with different concentration of cvNGF for 48 hours and counted. Each bar represents data of two independent experiments performed in triplicate; (**B**) The cells were incubated with 100 ng/mL cvNGF (black squares) or mNGF (red circles) during indicated period (2, 3, 4, or 5 day) and counted. Control cells were incubated without NGF (blue triangles). Each point represents data of two independent experiments performed in triplicate.

To check if cvNGF can directly affect breast cancer cells, we used MCF-7 cell line on which mNGF exerted proliferative effect [7,30]. In contrast to EAC which is solid or ascitic transplantable tumor, MCF-7 is a breast adenocarcinoma cell line widely used for *in vitro* cancer studies. It was found that cvNGF stimulated proliferation of MCF-7 cells in a concentration-dependent manner (Figure 4A), however the concentrations at which cvNGF induced noticeable cell proliferation were higher as compared to those described for mNGF [30]. The comparison of cvNGF proliferative effect with that of mNGF showed that the potencies of two factors in induction of MCF-7 cell proliferation were very close (Figure 4B). These results mean that the EAC growth inhibition by cvNGF cannot be explained by direct effect on cancer cells. It should be noted that suppression of subcutaneous tumor growth by mNGF was reported for nude mice implanted with DU145 prostate cancer cells [16]. The authors suggested that NGF prevents tumor growth via an indirect effect, probably innervation or maturation of the tumor neovasculature. We believe that the same mechanisms may be involved in EAC growth inhibition by cvNGF.

**Figure 5:**
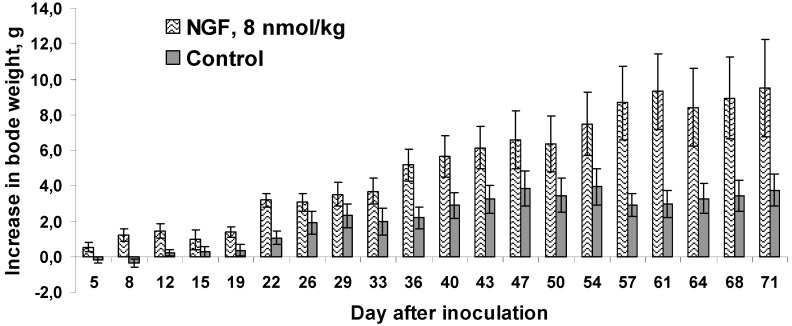
Gain in body weight by mice upon 19-day treatment with cvNGF. Mice were inoculated with 6 million EAC cells subcutaneously, in 15–20 min they received cvNGF intraperitoneally at dose of 8 nmol/kg body weight. Then, the injections were done intraperitoneally every 3–4 days 6 times during 19 days, *n =* 10. *p* (NGF-Control) < 0.01 as ANOVA for periods of 12 (when no difference in mean tumor size was registered between the test and control groups), 19 (during the course of cvNGF injections) and 71 days (the total time of observation).

The literature indicates that mNGF in addition to the important role in carcinogenesis affects body weight. Thus, the intraperitoneally injected mNGF reduced body weight gain in rats [31]. The same effect was observed in rats at chronic intraventricular mNGF administration [32]. It was also reported [33], that mNGF-treated newborn mice showed a slight, but significant, reduction in body weight gain. However, the opposite effect was observed in our experiments. In cvNGF-treated mice the increase in body weight gain was more pronounced as compared to mice in the control group (Figure 5). This was especially evident during the first week of treatment, when the control mice actually showed loss in body weight. Such an increase could not be explained by tumor growth, since it did not correlate with the increase in tumor volume, which was not different in treated and control animals up to the day 12 of experiment (Figure 2A). However, later on in the second half of the observation period the increase in body weight may be correlated with the tumor volume (Figure 2B and Figure 5). It should be noted that there are some data indicating that after brain damage NGF-treated rats ate more food and regained body weight more rapidly than control group [34]. The effect observed was explained by mNGF stimulation of the growth of regenerating neurons in the brain. This explanation is hardly true in our case. We have mentioned earlier that different organs express receptors for NGF and other neurotrophins [2]. Thus, it has been recently shown that exenatide, agonist of glucagon-like peptide-1 receptor, exerts its effects through the NGF/p75NTR system in mouse pancreas [35]. On the other hand, exenatide improves glycaemic control and stimulates satiety leading to reductions in food intake and body weight [36]. It might be possible that cvNGF disturbs the normal mechanisms involved in body weight maintenance and induce body weight gain.

## 3. Experimental Section

### 3.1. Materials

cvNGF was isolated from *Naja kaouthia* cobra venom as in [19]. The purity of samples was checked by analytical reversed-phase HPLC and MALDI mass spectrometry. It purity was 98%. mNGF was purchased from Life Technologies, Maryland, USA. Tyrosine kinase inhibitor K252a was from LC Laboratories, Woburn, MA, USA.

### 3.2. Mice

Female Af mice were inbreeded at the Institute of Physiology, National Academy of Sciences of Belarus (Minsk, Belarus). Ehrlich ascites carcinoma (EAC) was purchased from Blokhin Russian Cancer Research Center, Russian Academy of Medical Sciences (Moscow, Russia).

All the appropriate actions were taken to minimize discomfort to mice. World Health Organization’s International Guiding Principles for Biomedical Research involving Animals were followed during experiments on animals.

### 3.3. NGF Effect on Carcinoma Growth

Mice were inoculated with 6 million EAC cells subcutaneously on the back, in 15–20 min they received aqueous solution of cvNGF (0.8 μM in water) intraperitoneally at a dose of 8 nmol/kg body weight. Then, the injections were done intraperitoneally every 3–4 days 10 times during 32 days (experiment 1, *n =* 15) or 6 times during 19 days (experiment 2, *n =* 10). Control groups (*n =* 15 and *n =* 20 for the experiments №1 and №2, respectively) received distilled water.

Volume of developed subcutaneous tumor was assessed in living animals within 10 weeks of EAC cells injection by formula V = (a × b × c) × π/6, where V is tumor volume, cm^3^, a, b, c are diameters of tumor in three mutually perpendicular axes, cm, and π/6 ≈ 0,52 is a constant.

### 3.4. Combined K252a and NGF Effect on Carcinoma Growth

Mice grafted with 1 million EAC cells were randomly placed into seven groups: groups 1–3 (*n =* 8, *n =* 11 and *n =* 8) received only cvNGF at doses 4, 8, and 16 nmoles/kg, respectively; group 4 (*n =* 8) received only K252a (0.01% solution in dimethylsulfoxide (DMSO):water 1:20) at a dose 1 μg/g intraperitoneally; group 5 (*n =* 12) received K252a at dose similar to group 4 and then within 12–15 min cvNGF (8 nmoles/kg body weight) in saline. Control groups 6 (*n =* 9) and 7 (*n =* 13) received DMSO:water (1:20) and saline, respectively. For all groups the injections were done 10 times during 32 days (every 3–4 days).

### 3.5. Proliferation Assay on MCF-7 Cell Line

The human breast cancer MCF-7 cells were cultured in DMEM (with sodium pyruvate), supplemented with 2 mM L-glutamine, 10% FCS, 40 µg/mL gentamycine and 10 µg/mL insulin in a 5% CO_2_ incubator at 37 °C. The proliferation assay was performed generally as described [34]. Cells were plated at a density of 5 × 10^4^ in 35-mm plastic Petri dishes in a volume of 2 mL/dish. After 24 h, cells were wased twice with serum-free medium and the medium was replaced with 2 mL of fresh DMEM containing graded concentrations of NGF (10, 50, 100, and 200 ng/mL). When effect NGF as a function of time was studied, a concentration of 100 ng/mL was used. Cells were grown for 5 days and counted daily. Adherent cells were detached by rapid trypsinization. An adequate volume of medium containing trypan blue was added. Then cells were counted in automatic cell counter Countess. Experiments were performed in triplicate, and each experiment was repeated at least twice.

### 3.6. Statistical Analysis

Statistical analyses of the tumor volume were performed with nested design analysis of variance (ANOVA). Statistical analyses of the lifetime were performed with Mann-Whitney test. The differences were considered signiﬁcant for *P* values < 0.05. All results in figures presented as the mean ± SEM (standard error of the mean).

## 4. Conclusions

In summary, we have shown for the first time that cvNGF, a high-affinity ligand of TrkA receptor, suppresses *in vivo* the growth of EAC originating from breast cancer cells. However, this effect is short-term and reverses if course of cvNGF has stopped. Our data suggest that cvNGF slows down EAC growth via an indirect mechanism in which TrkA receptors are involved.
